# Clinical Outcome and Recurrence Risk of Chronic Subdural Hematoma After Surgical Drainage

**DOI:** 10.7759/cureus.35525

**Published:** 2023-02-27

**Authors:** Negar Atefi, Susan Alcock, Joseph A Silvaggio, Jai Shankar

**Affiliations:** 1 Department of Radiology, Max Rady College of Medicine, University of Manitoba, Winnipeg, CAN; 2 Department of Internal Medicine, Faculty of Health Sciences, University of Manitoba, Winnipeg, CAN; 3 Department of Neurosurgery, University of Manitoba, Winnipeg, CAN; 4 Department of Radiology, University of Manitoba, Winnipeg, CAN

**Keywords:** refractory subdural hematoma, chronic subdural hematoma, recurrence, surgical drainage, refractory subdural hemorrhage

## Abstract

Introduction

Chronic subdural hematoma (CSDH) is one of the most encountered neurosurgical cases. CSDH is defined as the accumulation of liquified blood products in the space between the dura and the arachnoid. A reported incidence of 17.6/100,000/year has more than doubled in the past 25 years in parallel with an aging population. Surgical drainage remains the mainstay of treatment, yet it is challenged by variable recurrence risks. Less invasive embolization methods of the middle meningeal artery (EMMA) could reduce the recurrence risks. Before adopting a newer treatment (EMMA), it is prudent to establish the outcomes from surgical drainage. The purpose of this study is to assess the clinical outcome and recurrence risk in surgically treated CSDH patients in our center.

Methods

A retrospective search of our surgical database was done to identify CSDH patients undergoing surgical drainage in the year 2019-2020. Demographic and clinical details were collected, and quantitative statistical analysis was performed. Peri-procedural radiographic information and follow-ups were also included as per the standard of care.

Results

A total of 102 patients (mean age: 69 years; range: 21-100 years; male: 79) with CSDH underwent surgical drainage with repeat surgery in 13.7% of the patients (n=14). Peri-procedural mortality and morbidity were 11.8%(n=12) and 19.6% (n=20), respectively. Overall, among our patient population, recurrence was seen in 22.55% (n=23). The mean total hospital stay was 10.6 days.

Conclusions

Our retrospective cohort study showed an institutional CSDH recurrence risk of 22.55%, in keeping with what is reported in the literature. This baseline information is important for a Canadian setting and provides a basis for comparison for future Canadian trials.

## Introduction

Globally, chronic subdural hematoma (CSDH) is one of the most encountered neurosurgical cases [1]. The incidence of CSDH is reported to be between 1.7 and 20.6 per 100,000 persons per year [2]. With an aging population and older age being a risk factor for CSDH, this number is only expected to increase. CSDH is expected to become the most treated cranial neurosurgical condition in adults by 2030 [3]. CSDH is a pathologic accumulation of old blood in the subdural space, which may cause effacement of brain structures leading to stroke-like symptoms or dementia [4,5]. The exact pathophysiology of CSDH is not yet fully understood. It has been suggested that CSDH is caused by a combined effect of minor trauma and inflammation processes, which can lead to neovascularization [6-8]. The frailty and leakiness of these new vessels can cause an ongoing hematoma in the subdural cavity [9,10]. The current treatment approaches for CSDH are divided into non-surgical and surgical strategies. Surgical strategies include twist drill craniotomy (TDC), burr hole drainage, and craniotomy or mini craniotomy. Burr hole drainage has been chosen as one of the standards of treatment for primary CSDH due to improved outcomes [11,12]. However, craniotomy is commonly the method of choice for recurrent hematomas [2,13]. Variable recurrence risk has been estimated to be between 7.5% and 29% for individuals suffering from CSDH [14-16]. To better understand and predict the risk of recurrence, many [4, 17-19] have attempted to develop risk factors and scoring systems to predict the risk of recurrence for these patients. These risk factors include, but are not limited to, older age (>75), bilateral hematoma operation, post-op pneumocephalus, antithrombotic use, and absence of a post-op drain [4,17-19]. A recent addition to non-surgical approaches in treating CSDH has been the embolization of the middle meningeal artery (EMMA). This endovascular technique targets the leakage of blood from the new vasculature and accommodates the rapid absorption of blood products into circulation [20]. Preliminary studies have been promising in reducing the risk of CSDH recurrence [14]. Randomized control trials are currently being conducted to evaluate the efficacy of EMMA [21]. Given the sub-optimal outcomes of surgical interventions and the emerging evidence for EMMA superiority when treating CSDH, the substitution or addition of this method as the standard of practice is on the horizon. Before offering a new treatment to reduce the risk of recurrence, it is essential to evaluate the safety and the risk of recurrences based on local surgical practices. Hence, the purpose of this retrospective cohort study is to provide baseline institutional efficacy (risk of recurrence) and safety (morbidity and peri procedural complications) in surgically treated CSDH patients.

## Materials and methods

The study was approved by our institutional research ethics board (REB# B2020:077) with a waiver of consent. This retrospective cohort study occurred at a tertiary care center, Health Sciences Center, Winnipeg, the provincial trauma, burn, stroke, and neurosurgical center. The sample included all consecutive patients from the Neurosurgery Department Operative list who underwent surgical drainage for subdural hematoma (SDH) in our institution between January 2019 and June 2020. The inclusion criteria were the symptomatic presence of CSDH on the CT head without contrast done immediately before surgery. Exclusion criteria were the presence of acute SDH, craniotomies with primary etiology other than an SDH (e.g., abscess drainage, tumor resection), unknown patients, or patients with missing information. Data was retrieved by performing chart audits and by reviewing images. Demographic and clinical data variables collected upon presentation to the hospital included: age; gender; whether admitted through the ED or neurosurgery clinic; history of trauma; use of antiplatelets and or anticoagulants; associated risk factors (hypertension, diabetes, stroke, renal disease, alcohol use, and smoking); presenting neurological symptoms; Glasgow coma scale on hospital presentation; baseline modified Rankin score (mRS) (at presentation); and whether the presentation was a primary or recurrent CSDH. Anticoagulation was either held or reversed before the surgery, to be resumed 48 hours after the intervention. The surgical method was fully at the discretion of the treating surgeon. The surgical data variables collected included the type of surgery (craniotomy vs. burr hole and if a drain was inserted) and the type of anesthesia used (general vs. conscious sedation). The patients were followed up as per the standard of care through in-person and/or virtual appointments. Functional status was obtained by using the mRS. The mRS was also collected 24 hours after surgery, at discharge, and on follow-up visits. Radiologic evaluation of CSDH was performed using the CT head without contrast. CSDH was defined when at least 50% of hematoma was either hypo- or iso-dense to grey matter. Baseline imaging data variables included the side of CSDH (left, right or bilateral), size of CSDH, cerebral atrophy score, and whether other images such as MRI or CT angiogram were done. The size of CSDH was recorded on the CT head without contrast done immediately pre-operative, immediately post-operative, and on follow-up, when available. The maximal thickness of CSDH was measured at the level of maximum axial thickness, and midline shift was measured on the axial slices. Cerebral atrophy score was determined as 0: normal volume; no ventricular enlargement; 1: opening of sulci/mild ventricular enlargement; 2: volume loss of gyri/moderate ventricular enlargement; and 3: ‘knife blade’ atrophy/severe ventricular enlargement. These measurements were recorded by a medical student (NA) under the guidance of an experienced interventional neuroradiologist (JS). Additional information (such as the presence of pneumocephalus, effacement of cortical sulci, etc.) and measurement in cases where images could not load were obtained from the clinical report.

Post-operative complications, mRS at discharge, discharge disposition, and total and post-operative length of hospital stay were recorded. In addition, the outpatient follow-up visits and hospital admission records were screened to record the changes in the mRS score. Due to the pandemic restrictions, the format of these visits shifted from in-person to over-the-phone visits. The primary study outcome was the safety and efficacy of surgically treated CSDH patients. Efficacy was measured in the recurrence rate of the CSDH. Recurrence of the symptoms, radiological information on the changes in the size of the hematoma, midline shift, and cerebral atrophy score were recorded at 30-day, 60-day, and 90-day time points when available. We considered ‘recurrence’ when there was returning/worsening of the symptoms and when radiological changes such as an increase in the size of the hematoma or midline shift were present. Safety outcomes were measured by the presence of any perioperative complications and mortality. The post-operative complications were recorded from the patient’s clinical chart. They included acute SDH, incision bleeding, myocardial infarction, pulmonary embolism, electrolyte abnormality, pneumonia, infection (wound, bone flap, subgaleal, etc.), and other negative outcomes. Morbidity was defined as an mRS>4 at discharge that resulted from a significant neurological complication post-surgery. This was due to the fact that several of these patients’ baseline mRS was up to 3. Secondary outcomes included a repeat CSDH evacuation surgery and in-hospital length of stay (LOS).

Statistical analysis

All statistical analyses were performed using the Stata 13.1 software (StataCorp, Texas, USA). The numbers of observations, mean, and SD were calculated for all continuous variables. Chi-squared tests were used for univariate analysis to test the association of categorical variables with peri-procedural morbidity, mortality, and recurrence of CSDH. Significant factors from the univariate analysis were included in the multivariate analysis using logistic regression to assess the association of the risk factors. A p-value of less than 0.05 was considered significant.

## Results

A total of 166 patients were categorized as undergoing SDH evacuation. Of these, 28 patients (23 with acute SDH from trauma; 3 with acute SDH from a prior neurosurgical intervention, one from prior stroke, 2 non-SDH primary cause of surgery, and one unknown patient) were excluded. Of the remaining 136 patients with CSDH confirmed on CT scan, 34 (25%) patients had no follow-up available and were not included in the final analysis. 

The final analysis was done on the remaining 102 patients (male-to-female ratio: 2.3; mean age of 69 years, range: 21-100). Clinical, imaging and demographic features of included patients are shown in Table 1. Among the 102 patients studied, 92% (n=94) had primary CSDH. According to the pre-operative radiological data, approximately 66% had unilateral, and only 34% had bilateral CSDH. The mean axial thickness of CSDH was measured at 19.09±6.81 mm on the right side and 15.4 ± 8.3 mm on the left side. Almost 75% (n=75) had a history of recent or remote trauma. Of these, 42 (41%) patients were on anticoagulation or antiplatelet medications.

**Table 1:** Demographic, pre-operative, and surgical information of patients with CSDH. CSDH: Chronic subdural hematoma; HTN: Hypertension; DM2: Diabetes Mellitus 2; CVA: Cerebrovascular accident; CKD: Chronic kidney disease; GCS: Glasgow Coma Scale; mRS: modified Rankin scale.

Parameter	Study population (n=102)
Sex Male Female	79 23
Age (year)	69±15.7
Presentation to hospital: Emergency Neurosurgery outpatient	99 2
History of trauma	75
Medication: Antiplatelet ASA 81 Other DAPT Anticoagulant Warfarin other	17 4 5 8 11
HTN	45
DM2	29
CVA	17
CKD	29
GCS on admission	13.29±2.96
Baseline mRS	1.29±1.35
Social history: Alcohol smoking	19 9
Type of CSDH Primary Recurrent	94 8
Side of CSDH Right Left Bilateral	33 33 34
Size right (mm)	19.09±6.81
Size left (mm)	15.4 ± 8.3
CTA Done	14
Cerebral Atrophy Score	0.62±0.79
Anesthesia General Conscious sedation Undocumented	71 30 1
Surgery Burr hole drainage Craniotomy Unknown	46 (37 with drain placement) 55 (52 with drain placement) 1
Periprocedural complications (all)	48
Peri-operative Morbidity (mRS 4)	20
In-hospital Mortality	12
Repeat surgery	14

Significant perioperative morbidity (mRS>4) was seen in 20 (19.6%) patients, and in-hospital mortality was seen in 12 (11.8%) patients. The average total and post-operative hospital stays were 10.6±11.5 days and 8.5±11.6 days, respectively. Forty-six patients had post-acute neurosurgical unit admission after initial discharge. On univariate analysis (Table 2), baseline mRS (p=0.006) and anticoagulation (p=0.005) were significantly associated with perioperative clinical complications. For multivariate analysis, age, sex, baseline mRS, and anticoagulation were included. Out of these, both baseline mRS (OR: 3.2; 95% CI: 1.2-8.9; p=0.022) and anticoagulation (OR: 3.3; 95% CI: 1.3- 8.3; p=0.013) were independently associated with perioperative clinical complications.

**Table 2:** Univariate analysis (using Chi-squared test) for risk factors associated with periprocedural morbidity, mortality and recurrence of CSDH. CSDH: Chronic subdural hematoma; mRS: modified Rankin score.

	Total (n)	Periprocedural morbidity (n)	P-value	Periprocedural mortality (n)	P-value	Recurrence (n)	P-value
Age <61 61-80 >80	26 56 20	4 11 5	0.54	2 6 4	0.39	4 14 5	0.59
Sex Male Female	79 23	16 4	0.94	9 3	0.82	23 0	0.03
Baseline mRS 0-2 >2	74 28	12 8	0.006	5 7	0.01	18 5	0.49
Anticoagulation Yes No	42 60	11 9	0.005	9 3	0.01	6 17	0.09
Laterality Right Left Bilateral	33 33 34	7 7 6	0.66	2 3 6	0.31	9 7 7	0.77
Primary Recurrent	94 8	20 0	0.38	11 1	0.86	22 1	0.48

On univariate analysis (Table 2), anticoagulation (p=0.01) and baseline mRS (p= 0.01) were significantly associated with perioperative mortality. For multivariate analysis, age, sex, baseline mRS, and anticoagulation were included. Of these, only anticoagulation (OR: 4.4; 95% CI: 1.1-18.2; p=0.043) was independently associated with perioperative mortality. The baseline mRS (OR: 3.5; 95% CI: 0.9-13.4; p=0.063) did not show a significant difference. On follow-up, 23 (22.5%) patients had a recurrence of CSDH. Some of the recurrences were picked up on follow-up scans (n=11), while others presented while being symptomatic (n=12) with stroke-like symptoms (weakness and facial droop), seizure, headache, dizziness, confusion, and agitation. Among those with recurrence, 14 (60.9%) patients underwent repeat surgery. On univariate analysis (Table 2), the male sex (p=0.03) was significantly associated with recurrence, and anticoagulation (p=0.09) showed some trend toward significance yet not statistically significant. All recurrences were seen in male patients only. For multivariate analysis, age, sex, baseline mRS, and anticoagulation were included. On multivariate analysis, anticoagulation (p=0.077) showed only a trend toward significance, and no statistically significant association was found.

## Discussion

In our retrospective cohort study of 102 patients with CSDH that were surgically treated only, we noticed that surgical drainage was associated with high morbidity and mortality. The perioperative morbidity and mortality were 19.6% (n=20) and 11.8% (n=12), respectively. Of these, 22.55% (n=23) had a recurrence of CSDH, and 61% (n=14) needed repeat surgical drainage. The high perioperative complications and mortality in our study are similar to what has been described in the literature to be between 0-25% and 0-32%, respectively [11,13,22]. These statistics also result in a high number (n=46) of post-acute neurosurgical unit admission after disposition and a longer patient recovery time. One of the major complications of CSDH surgical interventions is the recurrence of hematoma. The term ‘recurrence’ has been loosely defined in different studies and may cover resistance or incomplete resolution, rebleeds, or expanding hematomas [15]. There have been numerous reports on the recurrence risks of different centers, and results vary significantly, ranging from 0 to 76%, with a repeat surgery on approximately 10-20% of the cases [11,13,22]. Others [14-16] have found the recurrence risk to be estimated between 7.5% and 29% for individuals suffering from CSDH. Our institutional CSDH recurrence risk of 22.55% (n=23) and repeat surgery of 13.7% (n=14) fall within these reported ranges. Some of the recurrences were picked up on follow-up scans (n=11), while others presented while being symptomatic (n=12) with stroke-like symptoms (weakness and facial droop), seizure, headache, dizziness, confusion, and agitation. The reality that almost half the recurrences were asymptomatic and found on follow-up imaging highlights the importance of follow-up imaging. Our study found that among the CSDH patients with recurrence, 14 (60.9%) patients underwent repeat surgery. Undergoing repeat surgery further adds to the burden of care by lengthening LOS and health care costs. Furthermore, additional surgery increases the risk for patients. The significant variability in the risk of recurrence of CSDH after a surgical intervention that has been reported [14-16] causes discrepancies in findings and supports the need for a universal definition of ‘recurrence.’ More importantly, the high risk of recurrence reported in the literature supports the need for novel treatments for CSDH, such as EMMA. Our center uses both burr hole drainage (46% of cases) and craniotomy (55% of cases) when treating CSDH. Earlier reviews have shown that burr hole surgical drainage has better outcomes than craniotomy by having a more desirable cure-to-complications ratio [23]. In our study, the risk of mortality and recurrence was 14.5% and 32% for patients undergoing craniotomy compared to only 8.7% and 17.7% of patients receiving burr hole drainage treatment, respectively. Our findings are in keeping with what has been described in the literature. Our study confirmed the association between risk factors (baseline mRS and anticoagulation use) and the study's efficacy and safety measures. A higher mRS at baseline (p=0.022) and anticoagulant use prior to admission (p=0.013) were independently associated with peri-procedural clinical complications. Moreover, we found that anticoagulation (p=0.043) was associated with higher peri-procedural mortality, while a high baseline mRS demonstrated a trend (p=0.077) towards increased mortality. Previous studies have found an association between anticoagulation with a higher baseline mRS score and a less favorable outcome of surgery [2,24,25]. The use of anticoagulation showed a trend (univariate p=0.09 and multivariate p=0.077) toward significance for the recurrence risk of CSDH. Variable study results were found, with some confirming this association [2], while others found no association between the use of anticoagulation and the recurrence of CSDH [26,27].

Among the existing literature, a meta-analysis showed the recurrence risk of surgical drainage alone as 23.5% versus only 3.5% when surgical drainage was combined with EMMA, resulting in a 20% absolute risk reduction on-surgical intervention [14]. Using EMMA as the definitive treatment for CSDH is rapidly gaining attention, with multiple randomized trials designed to establish its superiority compared to traditional surgical interventions [28-31]. With a high risk of recurrence, 61% of our patient cohort needed a repeat surgical drainage. Repeat surgery has a higher complication rate than primary surgery and can be a predictive factor for mortality [32]. In a retrospective cohort study, the main reasons for repeat surgery after CSDH were reported to be acute post-operative hemorrhage, infections, and wound complications [15]. Hypertension and radiological factors such as midline shift and bilateral hematomas were reported as the strongest risk factors for repeat surgery. In addition, vitamin K antagonist anticoagulation use has been associated with a higher surgically relevant recurrence rate and repeat surgery. In our population of 102 patients, 33 were on some form of antiplatelet or anticoagulant medications, from which six (18.2%) had a recurrence of hematoma, and four (out of six) underwent repeat surgery. Although there are inherent limitations with a retrospective study design, since our study examined the efficacy and safety of CSDH patients treated with surgical drainage over a one-and-a-half-year time window, a retrospective design is well suited for this type of research. A large amount of observational data was gleaned from this review. However, the retrospective nature of this study is also the study’s biggest limitation. Study data were incomplete, leading to a smaller than excepted cohort. Loss to follow-up is another significant limitation of our study resulting in potential selection bias. However, our center covers a geographically unique part of the country, where a large population lives in remote rural areas with relatively limited access to healthcare. We believe that this was the main reason for the loss to follow-up in a significant proportion of our patients. The overall sample size was small, but we have presented consecutive patients with CSDH in our center. Furthermore, it provides baseline data on surgically treated CSDH at our institution.

## Conclusions

Our real-world observation suggested that the surgical drainage of CSDH results in high perioperative clinical complications and mortality, which is consistent with the existing literature. The risk of recurrence remains high (22.55%), with 61% of patients with recurrence needing repeat surgical drainage. This highlights the need for a novel treatment paradigm for patients with CSDH.
